# Reanalyzing the genetic history of Kra-Dai speakers from Thailand and new insights into their genetic interactions beyond Mainland Southeast Asia

**DOI:** 10.1038/s41598-023-35507-8

**Published:** 2023-05-24

**Authors:** Piya Changmai, Yutthaphong Phongbunchoo, Jan Kočí, Pavel Flegontov

**Affiliations:** 1 Department of Biology and Ecology, Faculty of Science, University of Ostrava, Ostrava, Czech Republic; 2 Department of Human Evolutionary Biology, Harvard University, Cambridge, MA USA; 3 Kalmyk Research Center of the Russian Academy of Sciences, Elista, Kalmykia Russia

**Keywords:** Anthropology, Population genetics

## Abstract

Thailand is a country where over 60 languages from five language families (Austroasiatic, Austronesian, Hmong-Mien, Kra-Dai, and Sino-Tibetan) are spoken. The Kra-Dai language family is the most prevalent, and Thai, the official language of the country, belongs to it. Previous genome-wide studies on Thailand populations revealed a complex population structure and put some hypotheses forward concerning the population history of the country. However, many published populations have not been co-analyzed, and some aspects of population history were not explored adequately. In this study, we employ new methods to re-analyze published genome-wide genetic data on Thailand populations, with a focus on 14 Kra-Dai-speaking groups. Our analyses reveal South Asian ancestry in Kra-Dai-speaking Lao Isan and Khonmueang, and in Austroasiatic-speaking Palaung, in contrast to a previous study in which the data were generated. We support the admixture scenario for the formation of Kra-Dai-speaking groups from Thailand who harbor both Austroasiatic-related ancestry and Kra-Dai-related ancestry from outside of Thailand. We also provide evidence of bidirectional admixture between Southern Thai and Nayu, an Austronesian-speaking group from Southern Thailand. Challenging some previously reported genetic analyses, we reveal a close genetic relationship between Nayu and Austronesian-speaking groups from Island Southeast Asia (ISEA).

## Introduction

Kra-Dai is a language family uniting about 90 languages spoken mainly in Southern China, Laos, Thailand, Vietnam, and Myanmar^1^. It is believed based on evidence from historical linguistics that this language family could have been spreading from Southern China to Southeast Asia since the eighth century CE^2^. The fact that hundreds of languages from five language families (Austroasiatic, Austronesian, Hmong-Mien, Kra-Dai, and Sino-Tibetan) are spoken in Mainland Southeast Asia (MSEA) reflects its ethnolinguistic diversity and complex population history. Limited genome-wide archaeogenetic studies previously suggested that some present-day Austroasiatic-speaking groups in MSEA are genetically similar to the first farmers who migrated to the region about four millennia before present^3,4^. Bronze-age individuals (dated to about two millennia before present) genetically similar to present-day Kra-Dai groups were found in Northern Vietnam^4^. This finding was interpreted as evidence of a migration wave of Kra-Dai speakers to Southeast Asia^4^. Thailand is located in the central part of MSEA. Thai, a language of the Kra-Dai family, is the official language of the country. Many other Kra-Dai languages and languages from the other four families mentioned above are also spoken in Thailand. Recent genome-wide studies^5,6^ on Thailand populations have shed light on the genetic population history of the country. We merged the data from both studies^5,6^ with other relevant published data, and re-analyzed them using new methods to scrutinize unexplored aspects of population history and re-examine previously proposed hypotheses. We focus on 14 Kra-Dai speaking groups from Thailand (Black Tai, Central Thai, Kalueang, Khonmueang, Khuen, Lao Isan, Lue (or Tai-Lue), Nyaw, Phuan, Phutai, Saek, Shan, Southern Thai, and Yuan) and five non-Kra-Dai-speaking control groups (Austroasiatic-speaking Bru, Khmu, and Palaung; Hmong-Mien-speaking Hmong Daw; and Sino-Tibetan-speaking Karen Padaung) along with other groups that exhibit genetic interactions with Kra-Dai-speaking populations, such as Nayu, a Malay(Austronesian)-speaking group from Southern Thailand. Our objectives are to investigate: (1) South Asian ancestry in the target groups; (2) their genetic structure and (3) genetic interactions with other populations in the region, which may be informative about routes of the migration of Kra-Dai speakers to the present-day Thailand territory; (4) test selected hypotheses on population history proposed in a previous study^5^.

## Results and discussion

### South Asian ancestry in Kra-Dai-speaking groups in Thailand

Indian (South Asian) influence has played a substantial role in shaping MSEA culture and history. Several ancient Indian-influenced states were located in various countries across Southeast Asia^7^. The earliest known Southeast Asian individual with South Asian ancestry is a child (78–234 calCE) from the Vat Komnou cemetery, Angkor Borei, Cambodia^8^, and various present-day Southeast Asian populations harbor a detectable amount of South Asian genetic ancestry^5,6,9^. Previous studies^5,6^ reported South Asian admixture in present-day populations from Thailand, namely Khmer, Kuy, Nyahkur, Mon, Central Thai, Southern Thai, and Nayu (Nayu, a Malay-speaking group from Southern Thailand, was labelled “SouthernThai_AN” in a previous study^5^; here we prefer the name “Nayu” as an endonym). Among these groups, only two populations, namely Central and Southern Thai, are Kra-Dai speakers.

To examine South Asian ancestry in the 14 Kra-Dai-speaking groups from Thailand listed above, we used three independent methods relying on different data types: autosomal haplotypes (*fastGLOBETROTTER*^10^), linkage disequilibrium (*ALDER*^11^), and allele frequencies (“admixture” *f*_*3*_-statistics^11^). Three Austroasiatic-speaking (Bru, Khmu, and Palaung), one Hmong-Mien-speaking (Hmong Daw), and one Sino-Tibetan-speaking group (Karen Padaung) were chosen as controls. Our findings are in line with those of Kutanan et al.^5^ in that we detected South Asian admixture in Central and Southern Thai using all three methods (Suppl. Tables 1–3). However, our analyses revealed that there are more populations in Thailand with detectable South Asian ancestry. The *fastGLOBETROTTER* analysis revealed South Asian admixture in two Kra-Dai-speaking (Khonmueang and Lao Isan) and in one Austroasiatic-speaking group (Palaung) (Suppl. Table 1). The presence of a South Asian genetic component in these populations was also supported by characteristic linkage disequilibrium decay curves (the *ALDER* method, Suppl. Table 2) and by significantly negative *f*_*3*_-statistics (for Khonmueang and Lao Isan) (Suppl. Table 3), or by linkage disequilibrium decay curves only (for Palaung) (Suppl. Table 2).

The *fastGLOBETROTTER* method found a one-date two-way admixture model (an East Asian + a South Asian proxy source) as best-fitting for Central Thai, Southern Thai, and Lao Isan, while a one-date multiple-way admixture model was best-fitting for Khonmueang and Palaung (Suppl. Table 1). The estimated admixture dates for Central and Southern Thai are around 800 years ago, which is similar to the dates previously estimated in Kutanan et al*.*^5^ using the same method. On the other hand, the admixture dates (95% confidence intervals) estimated for Lao Isan, Palaung, and Khonmeaung are 691–721, 603–664, and 359–386 years before present, respectively (Suppl. Table 1).

Admixture dates estimated by the *ALDER* method (based on plausible models with consistent linkage disequilibrium decay rates) are younger than those estimated by *fastGLOBETROTTER* for Central Thai and Palaung, but older in the case of Lao Isan (Suppl. Table 2). In the case of Central Thai and Khonmueang, the *fastGLOBETROTTER*-estimated dates fall within date ranges (across different source proxies) estimated by *ALDER* (see Suppl. Tables 1 and 2). We used broader sets of East Asian and South Asian surrogates for the *ALDER* as compared to the *fastGLOBETROTTER* analysis, and some plausible surrogates were absent in the latter analyses; that could explain the discrepancy in the estimated dates. Nonetheless, the *ALDER* results confirm all the findings of South Asian admixture by the *fastGLOBETROTTER* method.

### Inference of recent ancestry using *SOURCEFIND*

**Figure 1:**
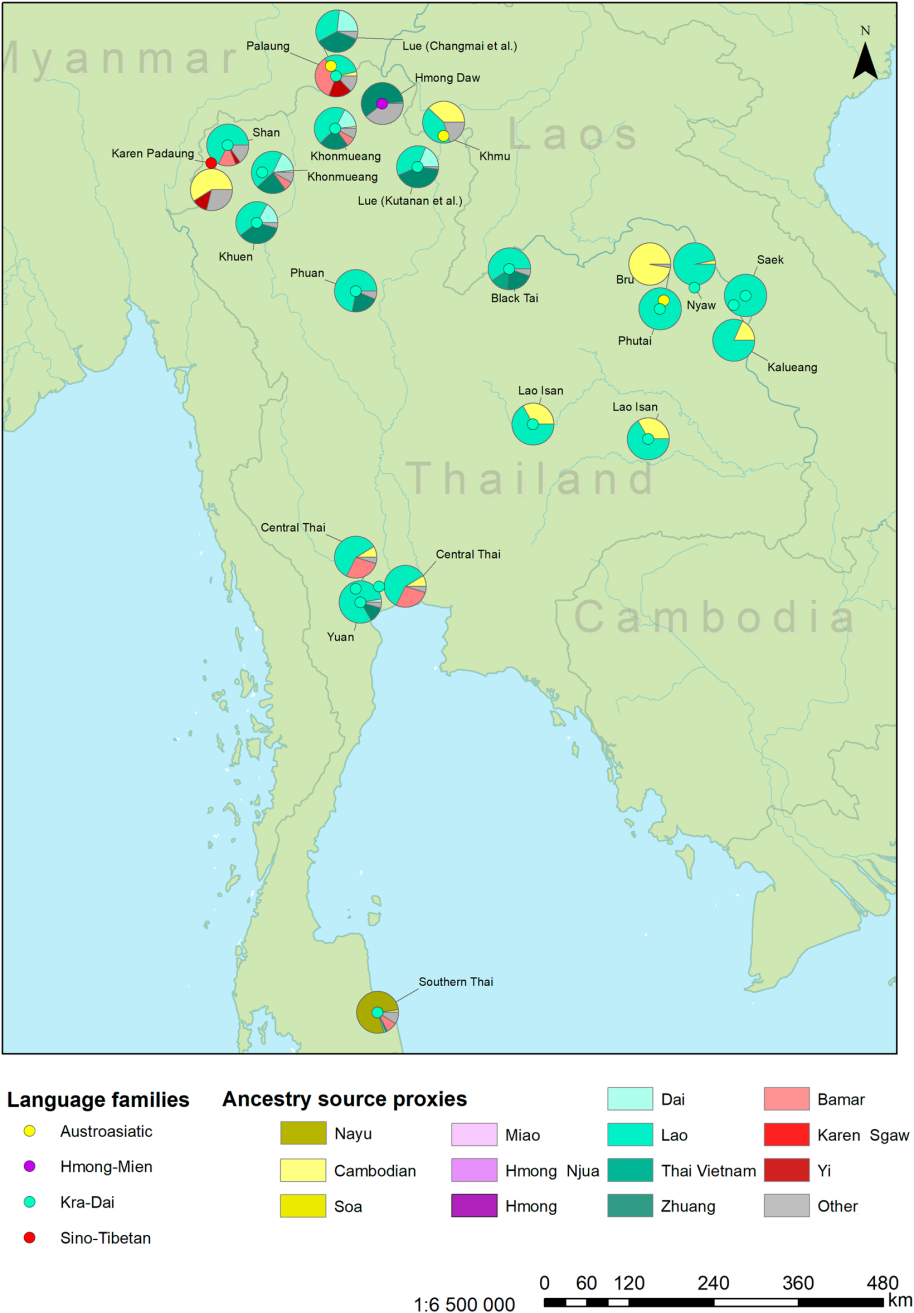
Sources of recent ancestry as inferred by *SOURCEFIND v.2* in groups from Thailand. Here only source proxies contributing > 10% to at least one target group are visualized (for full results see Suppl. Table 4). Locations of the groups on the map are shown with circles, and those are colored according to linguistic affiliation. Ancestry composition is illustrated with pie charts. Central Thai, Khonmueang, or Lao Isan individuals from multiple locations were merged into one group in this analysis. As a result, identical pie charts for Central Thai, Khonmueang, or Lao Isan appear across several locations on the map.

*SOURCEFIND*^12^ was introduced by the team that developed *ChromoPainter*^13,14^ and *GlobeTrotter*^13^, and unlike the latter software, which is mainly aimed at inference of admixture dates, *SOURCEFIND* is aimed specifically at inferring complex mixture models (proportions of genetic ancestry from multiple proxy sources). This tool implements a mixture model distinct from that used in *GlobeTrotter* and demonstrated better performance on simulated data^12^. For this reason, we decided to reanalyze the data reported by Kutanan et al.^5^ with *SOURCEFIND.* In this analysis we focused on the same set of populations analyzed by *fastGLOBETROTTER* above (14 Kra-Dai-speaking, one Hmong-Mien-speaking, one Sino-Tibetan-speaking, and three Austroasiatic-speaking groups, see Suppl. Table 4). Most other MSEA groups and selected East Asian and South Asian groups (Suppl. Table 4) were used as potential ancestry source proxies. Unlike *GlobeTrotter*, *SOURCEFIND* identifies source proxies whose contribution is distinguishable from noise and uses only those in constructing a mixture model. We show ancestry proportions for sources accounting for at least 1% of the genome in any target group in Suppl. Table 4, and ancestry proportions for major sources (> 10% of the genome in at least one group) in Fig. 1.

According to our mixture models (Fig. 1, Suppl. Table 4), a predominant ancestry component in most Kra-Dai speakers from Thailand is Lao-related (a Lao group from Laos was used as a source proxy). The fraction of Lao-related ancestry reached 95% in Kra-Dai speakers from the Northeast of Thailand near the Laos border. Bru, another group from Northeastern Thailand, demonstrates a strikingly different pattern, with < 1% of Lao-related ancestry (Fig. 1, Suppl. Table 4). Bru is a relatively isolated Austroasiatic-speaking group, and thus the patterns of recent ancestry inferred by *SOURCEFIND* are influenced not only by geography. The Lao-related ancestry component accounts for > 50% of ancestry in the Central Thai. In addition to Lao, we were able to trace genetic connections to Kra-Dai-speaking groups in Southern China: a Zhuang-related component accounted for up to 41% of ancestry in most Kra-Dai-speaking groups from Northern Thailand (Fig. 1, Suppl. Table 4). Remarkably, it was also detected at 11.5% in the Yuan group from Central Thailand who were resettled from Northern Thailand about 200 years ago^15^ (Fig. 1, Suppl. Table 4). The Black Tai (Tai Dam) group, which migrated from present-day Vietnam to the Loei province one to two centuries before present^16^, was modeled as having 13% of their ancestry from a Thai group in Vietnam. Nayu, an Austronesian-speaking group from Southern Thailand, contributes 66% of ancestry in Southern Thai, according to our model (Fig. 1, Suppl. Table 4). The genetic profiles of Southern Thai and Nayu are discussed further in the next section.

**Figure 2:**
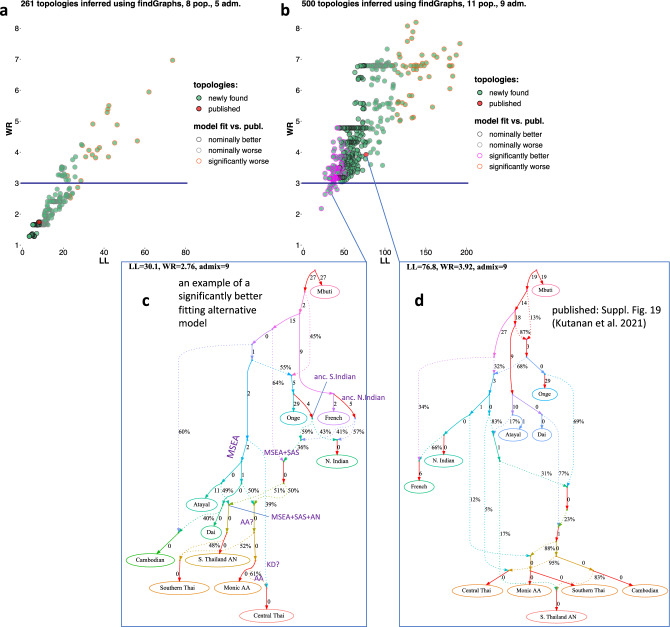
Fits of newly found and published^5^ admixture graph topologies to the published^5^ autosomal genetic data (the Human Origins SNP panel). Each distinct graph topology is visualized as a dot in the space of two model fit metrics: log-likelihood score (LL) and worst *f*-statistic residual (WR), with the published model highlighted in red. Results for the simpler complexity class (8 groups and 5 admixture events) are shown in panel (**a**), and results for the complex graphs (11 groups and 9 admixture events) are shown in panel (**b**). Results of model fit comparison tests on bootstrap replicates of the dataset^22^ are represented by different colors according to the legend. For example, models fitting significantly better than the published one are represented by circles with magenta outlines in panel (**b**). The fitted complex published model and its fit metrics (LL and WR) are visualized in panel (**d**), and an alternative complex model fitting the data significantly better and chosen as an example is shown in panel (**c**). Some edges of the alternative admixture graph can be interpreted as ancient populations attested or inferred in the archaeogenetic literature, and those are labeled in panel (**c**). Model parameters (admixture proportions and edge lengths measured in units of genetic drift) that cannot be estimated independently are highlighted in red. An algorithm for finding such unidentifiable parameters was introduced by Maier et al.^22^ The Nayu group is labelled as “S. Thailand AN” on the graphs, following Kutanan et al*.*^5^.

A surprising result of our inference of recent ancestry with *SOURCEFIND* is a large proportion of Bamar-related ancestry in the Central (24%) and Southern Thai (11%) groups (Fig. 2). This ancestry component was also detected in two groups (Palaung and Shan) located close to the border with Myanmar (Fig. 1), and in that case the result is correlated with geography. Our *SOURCEFIND* analysis (Suppl. Table 4), in contrast to *fastGLOBETROTTER* applied to the same *ChromoPainter* outputs*,* did not detect appreciable South Asian ancestry in the target groups, but we believe that the signal was obscured by the presence of groups with substantial South Asian admixture (Bamar and Cambodians) among the source proxies^6^.

Overall, the *SOURCEFIND* and *fastGLOBETROTTER* results suggest that the genetic profile of most Kra-Dai-speaking populations in Thailand is a result of admixture between Kra-Dai-speaking populations (from outside of Thailand) and Austroasiatic-speaking sources. The Lao groups from Laos were previously shown to harbor Austroasiatic-related ancestry^3,5^. Haplotype-sharing analyses in the latter study^5^ demonstrated a high level of genetic interaction between Khmu and Lao. This finding is consistent with our *SOURCEFIND* analysis in this study which estimated Lao-related ancestry in Khmu at 41% (Fig. 1, Suppl. Table 4). Despite the presence of Austroasiatic-related admixture in Lao, *f*_*4*_-statistics of the form *f*_*4*_ (Lao, Mbuti; a Kra-Dai group from China, an Austroasiatic-speaking group from MSEA) indicate that Lao is significantly closer to Kra-Dai-speaking groups from China than to Austroasiatic-speaking populations across Southeast Asia, including Kinh from Vietnam (Suppl. Figs. 1, 2).

Kra-Dai languages have roots in Southern China and were pushed gradually southward and then westward in the last few centuries BCE^17^. The violent purges that occurred in Southern China during the eighth to tenth centuries caused numerous small groups to migrate further south and west over many decades^17^. One of the known prominent routes is along the Mekong River^17^. A legend of Khun Borom, a mythical king who ruled and was believed to be the ancestor of Thai and Lao people in Southeast Asia, is thought to arise around the eighth century. The tale is regarded as evidence for actual emigration movements that occurred along the Tai people’s trade routes from Southern China to the present-day territories of Laos and Thailand^18,19^. The prominence of Lao-related ancestry in Kra-Dai-speaking groups from Thailand may suggest that Kra-Dai speakers migrated from Southern China to present-day Thailand via Laos. The combination of Dai- and Zhuang-related ancestry appearing only in some of our *SOURCEFIND* models for Kra-Dai speakers, specifically in Northern Thailand groups (Khonmueang, Lue, and Khuen) and in a group originating from that region (Yuan), suggests that an alternative migration route of Kra-Dai speakers to the present-day territory of Thailand might have existed. Strikingly, a scheme for the spread of Tai languages previously proposed by Baker and Phongpaichit^17^ places Lue, Khuen, and Yuan on the same route of southward migration that is distinct from other routes (see Map 1.3 in Baker and Phongpaichit^17^ for further details).

An alternative explanation for the omnipresence of the Lao-related genetic component in the Kra-Dai speakers from Thailand is an influx of Lao people to Thailand in the past. Indeed, there were several large-scale forced migrations triggered by wars between ancient Thailand and Laos. During the reign of King Taksin (1767–1782 CE), thousands of Lao people were relocated to Central Thailand. Later, in the beginning of the nineteenth century, following a Lao rebellion led by King Anouvong, about 150,000 Lao people were transported to Thailand, with about 50,000 moved to the Chao Phraya basin^17^, while the remainder were settled in Northeastern Thailand^20^.

### New insights into the genetic history of Central Thai, Southern Thai, and Nayu

An earlier study^21^, focused on the male-specific region of the Y chromosome (MSY), proposed an idea that the arrival of Kra-Dai languages in Thailand was an overwhelmingly cultural (not a demographic) process, due to a striking similarity of the Mon (local Austroasiatic-speaking) and Central Thai populations. A recent genome-wide study by Kutanan et al*.*^5^ suggested a close genetic relationship between Mon, Central and Southern Thai, and Nayu (labelled as “SouthernThai_AN”). It was concluded in that study^5^ that the genetic makeup of Nayu is distinct from Austronesian-speaking groups from Taiwan and Island Southeast Asia (ISEA) and that Nayu is genetically continuous with local Austroasiatic-speaking groups (implying that their Austronesian language was acquired via cultural transfer). As these conclusions were partly based on admixture graph results^5^, we revisited their admixture graph models using a new method, *findGraphs*^22^, which was not available at the time of their study.

Kutanan et al.^5^ presented two admixture graph models relevant for the conclusion above. The simpler model (in Fig. 6C^5^ of that study^5^) fitting the data well (with the worst-fitting *f*-statistic 1.6 standard errors away from the observed value) included Southern Thai and Nayu as groups cladal with Mon, and Central Thai got 22% of their ancestry from another East Asian source according to that model. The more complex model (in Suppl. Fig. 19^5^ of that study^5^) fitted the data poorly (the worst *f*-statistic residual was 4.1 SE) and was interpreted as largely supporting the simpler model. According to the complex model, Southern Thai, Central Thai, and Nayu are essentially cladal with two Austroasiatic-speaking groups (Mon and Cambodians) but differ from them slightly in the proportion of South Asian ancestry, or of Atayal-related ancestry in the case of Nayu. In other words, the sources of East Asian ancestry in Mon, Cambodians, and Thai are the same according to that model (see the complex published graph in Fig. 2d). These two admixture graph topologies were inferred automatically using the *AdmixtureBayes* tool^23^, and no alternative models with the same population composition and the same number of admixture events were shown or discussed.

We argue that using admixture graph models to support very specific statements about demographic history is an exercise that is fraught with problems on many levels. These conceptual problems are discussed in detail by Maier et al.^22^, where novel approaches for admixture graph inference are introduced. Here we revisited the same population sets and graph complexities (the number of admixture events allowed) that were used by Kutanan et al.^5^ and explored these spaces of graph topologies with an automated tool, *findGraphs*^22^. In other words, we attempted to find alternative well-fitting models for the simple and complex graphs presented by Kutanan et al.^5^, based on a very similar set of SNPs (Human Origins) and having the same population composition, the same complexity, and the same outgroup populations as in the original study. The *findGraphs* algorithm is seeded by a random graph of a given complexity and satisfying given constraints, and applies various graph-modification procedures iteratively, attempting to find a local optimum in the graph topology space (see Maier et al.^22^ for details). For each graph complexity class, the algorithm was started 500 times from random graphs and, for simplicity, only one inferred graph (best-fitting according to the log-likelihood score) was taken from each such run (see Methods for details). Fits of the resulting sets of distinct alternative topologies to the genetic data are visualized in Fig. 2.

The first problem of the admixture graph methodology becomes obvious when inspecting Fig. 2a,b: even a shallow exploration of the enormous spaces of alternative topologies reveals that dozens to hundreds of topologies fit the data approximately equally well (see also Maier et al*.*^22^). Two metrics are used in the literature for estimating fits of admixture graphs to *f*-statistic data, and those are worst *f*-statistic residuals (WR), also referred to as Z-scores and measured in standard errors (SE), or log-likelihood scores (LL) that take all *f*-statistics into account. Here we placed the newly inferred and the published models in the space of both metrics that are relatively well correlated (Fig. 2a,b).

Comparing fits of alternative admixture graph models in a statistically rigorous way is needed for any large-scale model exploration, and an algorithm for this purpose was introduced by Maier et al.^22^ The essence of this algorithm is fitting two alternative models on a set of bootstrap-resampled replicates of genetic data (this is easy to implement since for calculating SEs of *f*-statistics SNPs are divided into blocks based on genetic or physical distance) and comparing the resulting distributions of LL scores (see Maier et al.^22^ for details). Unlike previous methods for comparing fits of admixture graph models^24,25^, this method takes stochasticity in evolution of unlinked SNPs into account and makes no assumptions about the number of independent model parameters (which in the case of admixture graphs is not trivial to estimate)^22^. Relying on this bootstrap-based model comparison approach, we found that hundreds of alternative models (matching the simple or complex graphs in complexity) have fits to the data that are not significantly different from that of the published model (Fig. 2a,b) and that ca. 100 models fit the data significantly better than the complex published graph (Fig. 2b). We note that the same constraints on the graph topology were applied as in the original study: French or Mbuti were assigned as an outgroup. Since even a shallow exploration of both graph spaces found hundreds of models fitting the data as well as or significantly better than the published ones, deeper exploration of these topology spaces (performing more *findGraphs* runs and/or extracting more graphs from each run) is guaranteed to deliver further and further models of this kind^22^.

A question arises: is it justified to put a lot of weight on a particular graph and derive historical interpretations from its topology if hundreds of diverse topologies fit the data equally well? This is a key point discussed by Maier et al.^22^ when revisiting admixture graphs from eight published studies. For instance, it was found that there are at least several models fitting the data significantly better than the admixture graph for East Asians used to support a key conclusion by Wang et al.^26^, and the alternative models do not support the conclusion. These alternative models were found even after applying multiple topological constraints (guided by archaeology, linguistics, and other genetic studies) that Wang et al.^26^ relied on when constructing their graph model manually^22^.

What are the reasons for this high topological diversity among well-fitting models that was shown to be ubiquitous for admixture graph spaces explored in the literature^22^? First, there are inherent limitations of *f*-statistics related to directionality of gene flow: distinct graph topologies are known to yield identical *f*-statistics (see, for instance, Prüfer et al.^27^). Second, overfitting becomes a problem if too many admixture events are allowed. Overfitting was shown to be a common problem of admixture graphs reported in the literature^22^. Third, diversity of well-fitting topologies may result from a lack of reference populations that are differentially related to populations of interest and are needed for constraining the models^22^.

We believe that the latter point is especially relevant for interpreting the admixture graph results by Kutanan et al.^5^ We note that all the MSEA groups included in the published graphs (Cambodians, Mon, Central and Southern Thai, Nayu) are separated by very short genetic drift edges: the lengths of these edges are very close to 0 in the case of the simple (see Fig. 6C^5^ in the original study^5^) and complex (Fig. 2d) published graphs and all the alternative models we explored (see an example in Fig. 2c). This suggests that reference groups that could be instrumental in distinguishing the populations of interest (due to their differential relatedness to them) are lacking in the models. The simple eight-population graph is especially problematic in this respect since the only East Asian group outside of the region of interest (MSEA) in that analysis is Atayal from Taiwan, and no Tibeto-Burman-speaking or Kra-Dai-speaking proxies for potential ancestry sources were included. For instance, a Tibeto-Burman-related ancestry component was detected in Austroasiatic-speaking Mon by Kutanan et al.^5^ using methods other than admixture graphs, and also by Changmai et al.^6^ Given these results, the lack of a Tibeto-Burman reference population (and of other key reference groups) in both the simple and complex graphs from Kutanan et al.^5^ probably makes these admixture graph systems unconstrained. Therefore, it is not surprising that hundreds of simple topologies fit the data well in absolute terms (WR < 3 SE) and fit the data as good as the published model (Fig. 2a).

Since the complex graphs are more constrained than the simple ones (likely due to the inclusion of Kra-Dai-speaking Dai from Southern China), the alternative models we found are more differentiated according to their fits to the data (Fig. 2b). The published model on our dataset has a fit (WR = 3.9 SE) that is very close to that reported in the original study (WR = 4.1 SE), and such a fit is considered poor by convention since it exceeds 3 SE. We found 11 complex topologies (with 11 groups and 9 admixture events) that fit the data well in absolute terms (WR < 3 SE) and, moreover, significantly better than the published topology (with two-tailed empirical model-comparison *p*-values < 0.05). One such topology is shown as an example in Fig. 1c (model-comparison *p*-value = 0.002). As discussed in Maier et al.^22^, inference of demographic history in the admixture graph framework has important limitations even if best-practice protocols introduced in that study are adhered to. For instance, it is unknown what parsimony level (the number of admixture events) is optimal for inferring true history, and changing graph complexity can dramatically change the pattern of topologies that fit the data, and hence their historical interpretation^22.^ As discussed above, the outcomes of a model inference protocol also depend a lot on the choice of groups included in the model^22^. For these reasons we did not believe that any well-fitting model found by us is accurate; on the contrary, we believe that all of them are wrong in one way or another. However, the model shown in Fig. 1c has several features that match archaeogenetic results reported in the literature and derived using various methods other than admixture graphs: (1) Indians are derived from Ancient North Indians of West Eurasian origin and Ancient South Indians related to the Andamanese^28,29^; (2) there is a fraction of Atayal (Austronesian)-related ancestry in Nayu from Southern Thailand, who are Austronesian speakers^5,6^; (3) there is South Asian (Indian) ancestry in nearly all MSEA groups included in the model^5,6^.

Importantly, the alternative model shown in Fig. 2c (and other alternative models we found) is in concordance with our *SOURCEFIND* results (Fig. 1, Suppl. Table 4). If we interpret the graph node marked as “MSEA + SAS” (Fig. 1c) as an Austroasiatic-speaking group with Indian admixture similar in its ancestry composition to Mon^5,6^, then the Nayu group gets 49% of their ancestry from an Austronesian Atayal-related source, Southern Thai get 48% of their ancestry from Nayu, and Central Thai get 39% of their ancestry from an unidentified East Asian source (Fig. 2c). We stress again that although the alternative model presented in this study fits the data well and significantly better than the published one, we do not claim it to be fully accurate.

**Figure 3:**
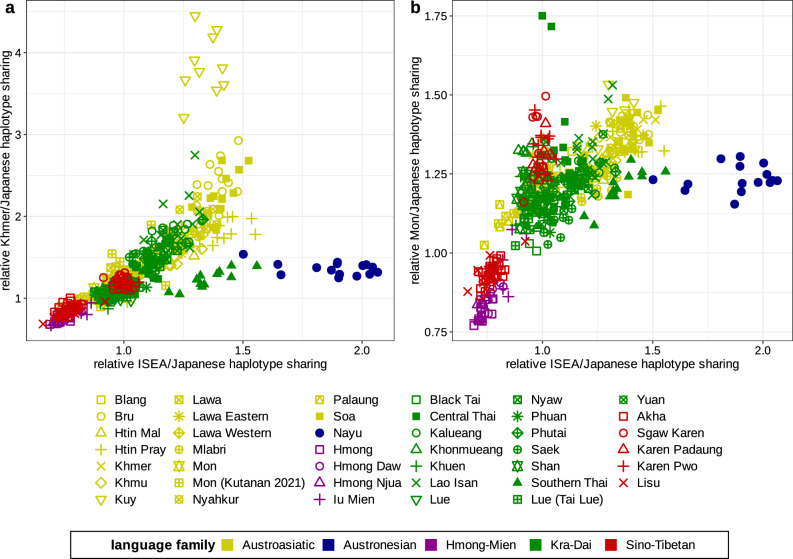
Scatterplots illustrating (**a**) relative ISEA/Japanese haplotype sharing vs. relative Khmer/Japanese haplotype sharing statistics and (**b**) relative ISEA/Japanese haplotype sharing vs. relative Mon/Japanese haplotype sharing statistics. The ISEA group is composed of four populations from ISEA (Barito, Dusun, Murut, and Semende). Each point in the plot represents an individual. Language families are color-coded according to the legend.

According to the *SOURCEFIND* method, the Southern Thai group was modelled as having around 65% of their ancestry derived from Nayu (Fig. 1, Suppl. Table 4). Therefore, for understanding the genetic origin of Southern Thai, a good model for the genetic history of Nayu is needed. As mentioned above, Kutanan et al*.*^5^ suggested that Nayu (Austronesian speakers) are genetically continuous with local Austroasiatic-speaking groups in the MSEA region. To gain further insight into the genetic relationship between Nayu and other Austronesian-speaking groups (from Taiwan and ISEA), we first tested whether the Nayu group is genetically closer to Austroasiatic-speaking groups from MSEA or Austronesian-speaking populations from ISEA. *f*_*4*_-statistics of the form of *f*_*4*_ (Nayu, Mbuti; ISEA, AA), where “ISEA” is an Austronesian-speaking group from ISEA and “AA” is an Austroasiatic-speaking group from Thailand and Cambodia, show significantly positive values (Z-scores above 2 in most cases, Suppl. Fig. 3), indicating that Nayu share more genetic drift with the former populations. We further inspected scatterplots illustrating for various populations from Thailand relative autosomal haplotype sharing with Austroasiatic-speaking populations (Mon or Khmer) and with merged Austronesian-speaking groups from ISEA (Barito, Dusun, Murut, and Semende) (Fig. 3). While Nayu individuals show levels of the “Austroasiatic” haplotype sharing statistic that are average for the populations from Thailand, they demonstrate the highest levels of the “Austronesian” haplotype sharing statistic in Thailand (Fig. 3). Southern Thai also have elevated “Austronesian” haplotype sharing statistics, although to a lesser extent (Fig. 3). This finding is consistent with our *SOURCEFIND* (Fig. 1, Suppl. Table 4) and admixture graph results (Fig. 2), which reveal a substantial amount of gene flow from Nayu to Southern Thai. It is noteworthy that in Fig. 3a, Austroasiatic-speaking Kuy individuals are clearly outliers according to the “Khmer” haplotype sharing statistic. This finding is in line with previous research in linguistics^30^ and genetics^6^, which proposed that Kuy and Khmer have had prolonged interactions and share a similar genetic makeup.

**Figure 4:**
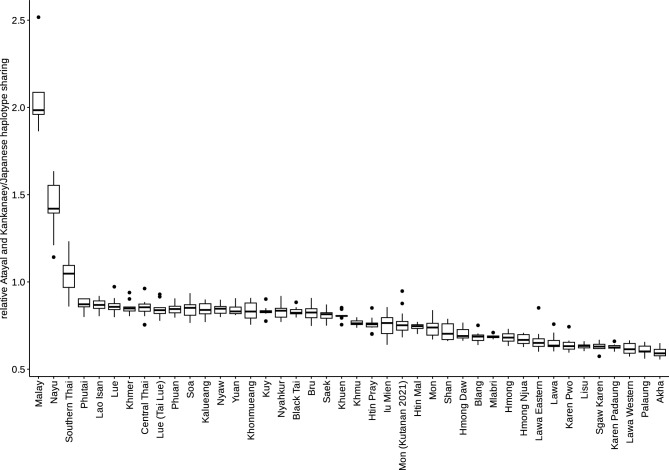
Box plots illustrating distributions of relative (Atayal and Kankanaey)/Japanese haplotype sharing statistics in populations from Thailand. Atayal and Kankanaey were grouped together to represent Austronesian speakers from a location that is close to the putative homeland of the language family^32^.

Previous studies reported the presence of an Austroasiatic-related genetic component in present-day Austronesian-speaking groups^3^ and in ancient individuals from ISEA^31^. In the light of these results, we sought to investigate whether Nayu also harbor genetic ancestry in common with populations close to the putative homeland of Austronesian languages^32^, such as Atayal and Kankanaey (Austronesian-speaking groups from Taiwan and Northern Philippines), which probably have a minimal level of Austroasiatic-related ancestry^33^. Most Nayu and Southern Thai individuals indeed exhibit a higher level of haplotype sharing with Atayal and Kankanaey than all the other individuals from Thailand (Fig. 4). A Malay group from Singapore was used as a positive control. These findings also support the presence of high levels of Nayu admixture in Southern Thai inferred by *SOURCEFIND* (Fig. 1, Suppl. Table 4). The lower level of Atayal and Kankanaey haplotype sharing in Nayu as compared to Malay from Singapore suggests that Nayu could have possibly admixed more with non-Austronesian speaking populations, which indicates genetic diversity among Malay-speaking groups.

We further infer the recent ancestry of Nayu using *SOURCEFIND* (Suppl. Table 5). Southern Thai emerged as the most prominent surrogate, accounting for approximately 57% of Nayu’s ancestry (Suppl. Table 5) and suggesting bidirectional gene flow between Southern Thai and Nayu. A previous study based on short tandem repeats showed a similar result^34^. Additionally, up to 40% of Nayu’s ancestry was derived from a Malay group as a surrogate. This extensive bidirectional gene flow between Southern Thai and Nayu may obscure signals of genetic ancestry from other groups. Considering this concern, we repeated the *SOURCEFIND* analysis for Southern Thai and Nayu, but not allowing these groups to be haplotype donors for each other. When we removed Southern Thai from the list of donors for Nayu, *SOURCEFIND* infers Malay from Singapore as the most prominent source, followed by Cambodian and Lao (59%, 14%, and 9%, respectively) (Suppl. Table 5). This finding suggests that the correct model for Nayu probably includes Austroasiatic- and Kra-Dai-related ancestry, in addition to Austronesian-related ancestry. Likewise, a *SOURCEFIND* analysis without Nayu as a donor for Southern Thai inferred Lao and Malay (from Singapore) as the most prominent surrogates (accounting for 23% and 22%, respectively), with high levels of Cambodian- and Bamar-related ancestry (14% and 15%, respectively) (Suppl. Table 5). All in all, the result shows that Southern Thai harbor Kra-Dai-related and Austroasiatic-related ancestry as the other Kra-Dai-speaking groups from Thailand we re-analyzed. However, the high level of Austronesian-related ancestry in Southern Thai makes this group unique among Kra-Dai-speaking populations from Thailand.

The study by Kutanan et al*.* also suggested a close genetic relationship, “genetic continuity or admixture”, between Central Thai and Mon^5^. However, our analyses do not find any clear evidence for this. Statistics *f*_*4*_ (Mon, Mbuti; Central Thai, another group from Thailand) (Suppl. Fig. 4) and *f*_*4*_ (Central Thai, Mbuti; Mon, another group from Thailand) (Suppl. Fig. 5) suggest that the Mon group shares significantly more genetic drift with most other groups from Thailand than with Central Thai; and Central Thai also shares significantly more genetic drift with most other groups from Thailand than with Mon (Suppl. Figs. 4, 5). Nevertheless, we caution that complex genetic history of Mon and Central Thai may affect *f*_*4*_-statistics, and the results do not entirely dismiss the possibility of a close genetic relationship between Mon and Central Thai. We found that two Central Thai individuals (CT205 and CT217) demonstrate haplotype sharing with Mon that is unusually high among all the other populations from Thailand we re-analyzed (Fig. 3b). These individuals do not demonstrate elevated haplotype sharing with Austroasiatic-speaking Khmer (Fig. 3a). Notably, individuals CT205 and CT217 are from the same location (Photharam district, Ratchaburi province). A major city of Dvaravti, a putatively Mon-speaking state which existed in the seventh to eleventh centuries CE^35^, had been located in the Ratchaburi province^36^, and the Photharam and Ban Pong districts in the Ratchaburi province are among present-day centers of the Mon ethnic group in Thailand^15^. It is therefore not surprising that some Central Thai individuals from this region have inherited Mon genetic ancestry. Other Central Thai individuals from Potharam and other locations do not exhibit elevated haplotype sharing with Mon as they cluster among other populations from Thailand (Fig. 3b).

## Conclusions

In this study, we report new insights into the genetic history of Kra-Dai-speaking and other human populations from Thailand. We revealed South Asian admixture in Kra-Dai-speaking Lao Isan and Khonmueang, and Austroasiatic-speaking Palaung; South Asian admixture in these populations was not observed in a previous study^5^. All our results also indicate that genomes of Kra-Dai-speaking populations from Thailand analyzed in this study are best modelled as a mixture of Kra-Dai-related and Austroasiatic-related ancestry. This finding supports the admixture scenario for the spread of Kra-Dai speakers to Thailand: Kra-Dai-speakers, who originated in Southern China, migrated to present-day Thailand and subsequently inter-married with local Austroasiatic-speaking populations. The fact that Lao emerged as the most prominent ancestry surrogate for most Kra-Dai speakers from Thailand indicates that present-day Laos could have been an important gateway for this migration. The presence of Dai and Zhuang ancestry in some groups from Northern Thailand (Khonmueang, Khuen, and Lue) and a group which migrated recently from that region (Yuan) points to an alternative route for the spread of some Kra-Dai languages. These two routes agree with a historical linguistic scenario proposed by Baker and Phongpaichit^17^ (see Map 1.3 in that study). Our study suggests a rich genetic history of Thailand, with several ancestry components correlated with geography and/or linguistic affiliation. We demonstrated bidirectional admixture between Southern Thai and Nayu, an Austronesian-speaking group from Southern Thailand, and a close genetic relationship between Nayu and Austronesian-speaking groups from Island Southeast Asia, which suggests a migration of Austronesian speakers from Island Southeast Asia to Mainland Southeast Asia. The Austronesian-speaking migrants then admixed with local Mainland Southeast Asians, probably Austroasiatic and Kra-Dai speakers. Our results do not support genetic continuity or particularly close genetic connection between Mon and Central Thai, except for two Central Thai individuals from the Potharam district of Ratchaburi province who show a level of haplotype sharing with Mon that is unusually high for other populations from Thailand. In contrast, such an ancestry profile is not observed for the other Central Thai individuals from the same and different locations.

## Methods

### Assembling the dataset

Previously collected DNA samples for 10 present-day Lao individuals^37,38^ were provided through the curtesy of Kenneth Kidd and Judith Kidd. No human subject was directly involved in this study. The samples were made anonymous before being sent to the Kidd laboratory^37^. The samples were collected with informed consent under a protocol approved by the IRB at Yale University^38^ which was also reviewed and approved by the NIGMS (National Institute of General Medical Sciences of the U.S. National Institute of Health) and by CEPH (Center for the Study of Human Polymorphisms in Paris)^37^. Each of the subjects provided consent for the collection of samples and their utilization in general population studies^37^. All experiments were performed in accordance with relevant guidelines and regulations. The DNA samples were genotyped on the Affymetrix Human Origins SNP array^39^ at the Reich Lab (Harvard University, MA, USA), and David Reich provided the new genotype data for this study as a courtesy. We merged the newly generated data for Lao with published diploid genotyping data generated on the Affymetrix Human Origins SNP array^9^ mainly in the following studies: Kutanan et al.^5^, Liu et al.^40^, Wang et al.^26^, Changmai et al.^6^, Lazaridis et al.^41^, Nakatsuka et al.^42^ For a list of individuals, groups, their linguistic affiliations, and data sources see Suppl. Table 6. All our work relied on a set of 574,131 autosomal SNPs from the Human Origins panel identical to that used in Changmai et al.^6^.

### Exploring admixture graph topology spaces

All our work with *f*-statistics and methods relying on them was done using the *ADMIXTOOLS 2* package^22^ (https://uqrmaie1.github.io/admixtools/). To calculate *f*-statistics needed for fitting admixture graph models, we first used the “*extract_f2*” function with the “*maxmiss*” argument set at 0, which corresponds to the “*useallsnps: NO*” setting in the classic *ADMIXTOOLS*^39^. It means that no missing data are allowed (at the level of populations) in the specified set of populations for which pairwise *f*_*2*_-statistics are calculated. The “*blgsize*” argument sets the SNP block size in Morgans, and we used the default value of 0.05 (5 cM). Since all groups involved in the admixture graph modelling in this study included more than one individual, and diploid variant calls were available for all individuals, the “*adjust_pseudohaploid*” and “*minac2*” arguments were set to “*FALSE*”^22^. The “*extract_f2*” function calculates *f*_*2*_-statistics for all pairs of groups per each SNP block, and those are used by the “*find_graphs*” and “*qpgraph*” functions for calculating *f*_*3*_- and *f*_*4*_-statistics as linear sums of *f*_*2*_-statistics^39^. In the absence of missing data, the linear sums should be unbiased^22^.

We used the “*find_graphs*” function from the *ADMIXTOOLS 2* package for finding alternative well-fitting admixture graph topologies. We worked on the sets of groups and individuals that were identical to those used by Kutanan et al.^5^ for constructing their admixture graphs presented in Fig. 6C^5^ (8 groups and 5 admixture events) and Suppl. Fig. 19^5^ (11 groups and 9 admixture events). We did not modify the graph complexity (the number of admixture events) either and kept the outgroups used by Kutanan et al.^5^: French for the simpler graph and Mbuti for the complex graph. Characteristics of the datasets used for admixture graph fitting in our study are as follows: (1) graphs of the “simple” complexity class were based on 8 groups, 177 individuals (Suppl. Table 6), and 456,719 sites polymorphic in this set of groups and having no missing data at the group level; (2) graphs of the “complex” class were based on 11 groups, 207 individuals (Suppl. Table 6), and 501,703 sites polymorphic in this set of groups and having no missing data at the group level.

For each graph complexity class, the *findGraphs* algorithm was started 500 times independently, seeded by random graphs with a specified number of admixture events (5 or 9) and a specified outgroup (French or Mbuti). Random graphs were generated using the “*random_admixturegraph*” function. The settings of the *findGraphs* algorithm were identical to those presented in Maier et al.^22^ (see Appendix 1, Section 2 in that study), and French or Mbuti were specified as outgroups at this topology optimization step too. From each *findGraphs* run, one best-fitting topology (i.e., the highest-ranking topology according to the log-likelihood score) was extracted, and a set of non-redundant topologies was constructed from all the 500 runs. Fits of these topologies to the data (log-likelihood scores and the worst *f*-statistic residuals) were plotted, and best-fitting topologies were inspected manually for features that are important for historical interpretations. The published admixture graph topologies (Fig. 6C^5^ and Suppl. Fig. 19^5^ from Kutanan et al.^5^) were fitted to the same per-block *f*_*2*_-statistic data (that were used for inference of new fitting admixture graphs) using the “*qpgraph*” function with the following settings: “*numstart* = *100, diag* = *0.0001, return_fstats* = *TRUE*”.

To find out if newly found admixture graph models fit the data significantly better or worse than the published ones, we used a bootstrap-based model comparison algorithm developed by Maier et al.^22^ Five hundred bootstrap replicates of the two SNP block datasets, corresponding to the simple and complex published graphs, were generated (with the 5 cM block size). The algorithm reports empirical two-tailed *p*-values; 0.05 was used as a statistical significance threshold; and the settings of the algorithm were identical to those used by Maier et al.^22^.

### Methods based on autosomal haplotypes

We phased a world-wide dataset of 3945 individuals (compiled from published sources) using *SHAPEIT v.2 *(*r900*)^43^ with a 1000 Genomes Phase 3 genetic map^44^. We then ran *ChromoPainter v.2*^13,14^ to generate inputs for *SOURCEFIND v.2*^12^ and *fastGLOBETROTTER*^10^. We selected 75 surrogates and 20 target populations (14 Kra-Dai-speaking groups from Thailand for whom the data were reported by Kutanan et al.^5^, one Kra-Dai-speaking group from Thailand for whom the data were reported by Changmai et al.^6^, and five control groups from Thailand speaking other languages); see a list of populations involved in Suppl. Table 1 or Suppl. Table 4. We ran *ChromoPainter v.2* assigning all the surrogates as donors and recipients, but the target populations were assigned as recipients only. This means that target populations receive haplotypes only from surrogates, but not from their own population nor other target populations. The other details of the *ChromoPainter v.2* protocol exactly followed those presented by Changmai et al.^6^.

The settings of the *SOURCEFIND* algorithm used for inferring complex mixture models for the same set of 20 target groups were identical to those used by Changmai et al.^6^, and all the 75 surrogates were used as a panel of potential sources from which the algorithm constructed mixture models for each target. The settings of the *fastGLOBETROTTER* algorithm were also identical to those used by Changmai et al.^6^, and all the 75 surrogates were used for inferring best-fitting admixture models and estimating admixture dates.

For generating the *SOURCEFIND* results in Suppl. Table 5, we used different lists of donor and recipient populations for *ChromoPainter v.2* (see the complete lists in Suppl. Table 5). In these particular *SOURCEFIND* analyses, we removed the target (Nayu or Southern Thai) from the list of surrogates, but assigned all the other populations as surrogates. Here is a brief explanation of the dataset compositions for these analyses:

1. List 1, all 95 groups (75 surrogates + 20 targets from the previous setup) were assigned as both donors and recipients for a *ChromoPainter v.2* analysis. Using this set of populations, we aimed to test a hypothesis proposed in a previous study^5^, which suggested that the Nayu group (Austronesian-speaking) is genetically continuous with Austroasiatic-speaking groups. In other words, we examined if Nayu individuals’ genomes can be modelled as derived exclusively (or mostly) from Austroasiatic-speaking groups.
2. List 2, 94 groups from list 1 (all except for Southern Thai) were assigned as both donors and recipients for a *ChromoPainter v.2* analysis. As we demonstrated bidirectional admixture between Nayu and Southern Thai, the large proportion of Nayu genomes derived from a Southern Thai-related group may obscure signals of other ancestry components in Nayu. Therefore, we performed a *ChromoPainter v.2* analysis again, excluding Southern Thai from the dataset, which allows more haplotype sharing between Nayu and the other groups.
3. List 3, 94 groups from list 1 (all except for Nayu) were assigned as both donors and recipients for a *ChromoPainter v.2* analysis. Due to the bidirectional admixture between Southern Thai and Nayu, the high level of Nayu-related ancestry in the Southern Thai group may mask signals of other ancestry component in Southern Thai. Thus, we performed a *ChromoPainter v.2* analysis again excluding Nayu from the dataset, which allows more haplotype sharing between Southern Thai and the other groups.

For calculating relative haplotype-sharing statistics, we first performed a *ChromoPainter v.2* analysis on 95 populations from list 1 with the “-a” argument, which instructs the software to paint each recipient individual using all the other individuals as donors. We followed a method for computing relative haplotype-sharing statistics described by Flegontov et al*.*^25^. In brief, we calculated the average haplotype-sharing statistic between individual *A* and population *B* (*aHSS*_*AB*_) by (1) summing up the genetic length of the DNA that individual *A* copied from an individual in population *B* (individual *B*_*j*_) as well as the DNA length copied in the opposite direction (from *B*_*j*_ to *A*), which yields the haplotype-sharing statistic between individuals *A* and *B*_*j*_ (*HSS*_*ABj*_); (2) averaging *HSS*_*ABj*_ across all individuals in population *B* (*aHSS*_*AB*_). We then normalized *aHSS*_*AB*_ by the average haplotype-sharing statistic between individual *A* and a Japanese group (*aHSS*_*AJapanese*_) to estimate relative haplotype sharing between individual *A* and population *B*. In short, the relative haplotype-sharing statistic between individual *A* and population *B* = *aHSS*_*AB*_/*aHSS*_*AJapanese*_. For example, relative haplotype-sharing between individual CT201 (Central Thai) and the Khmer group = *aHSS*_*CT201Khmer*_*/aHSS*_*CT201Japanese*_.

### Fitting admixture models to linkage disequilibrium decay curves

We used the *ALDER* tool^11^ with the default settings for fitting two-way admixture models of the type “East or Southeast Asian group + South Asian group” for the set of 20 target groups. For a list of groups involved see Suppl. Table 2.

### f-statistics

*f*_*3*_*-statistics* and *f*_*4*_*-statistics* were calculated using the “*qp3pop*” or “*qpdstat*” functions of the *ADMIXTOOLS 2* package^22^. Both *f*_*3*_*-statistics* and *f*_*4*_*-statistics* were calculated directly from genotype data, without *f*_*2*_-statistics as an intermediate. For each triplet or quadruplet of groups, no missing data were allowed at the group level (the default setting).

## Supplementary Information

Supplementary Figure 1.

Supplementary Figure 2.

Supplementary Figure 3.

Supplementary Figure 4.

Supplementary Figure 5.

Supplementary Table 1.

Supplementary Table 2.

Supplementary Table 3.

Supplementary Table 4.

Supplementary Table 5.

Supplementary Table 6.

## Data Availability

The newly generated genome-wide genotype data for 10 Lao individuals is publicly available at the Reich Lab website (https://reich.hms.harvard.edu/datasets) and is also available at 10.5281/zenodo.7896155.
