# Supplementary material for: Reanalyzing the genetic history of Kra-Dai speakers from Thailand and new insights into their genetic interactions beyond Mainland Southeast Asia
Source: Sci Rep. 2023 May 24;13:8371. doi: 10.1038/s41598-023-35507-8 (PMC10209056; doi:10.1038/s41598-023-35507-8)
Supplement: Supplementary file 1 — Supplementary Figure 1. [file 41598_2023_35507_MOESM1_ESM.pdf]

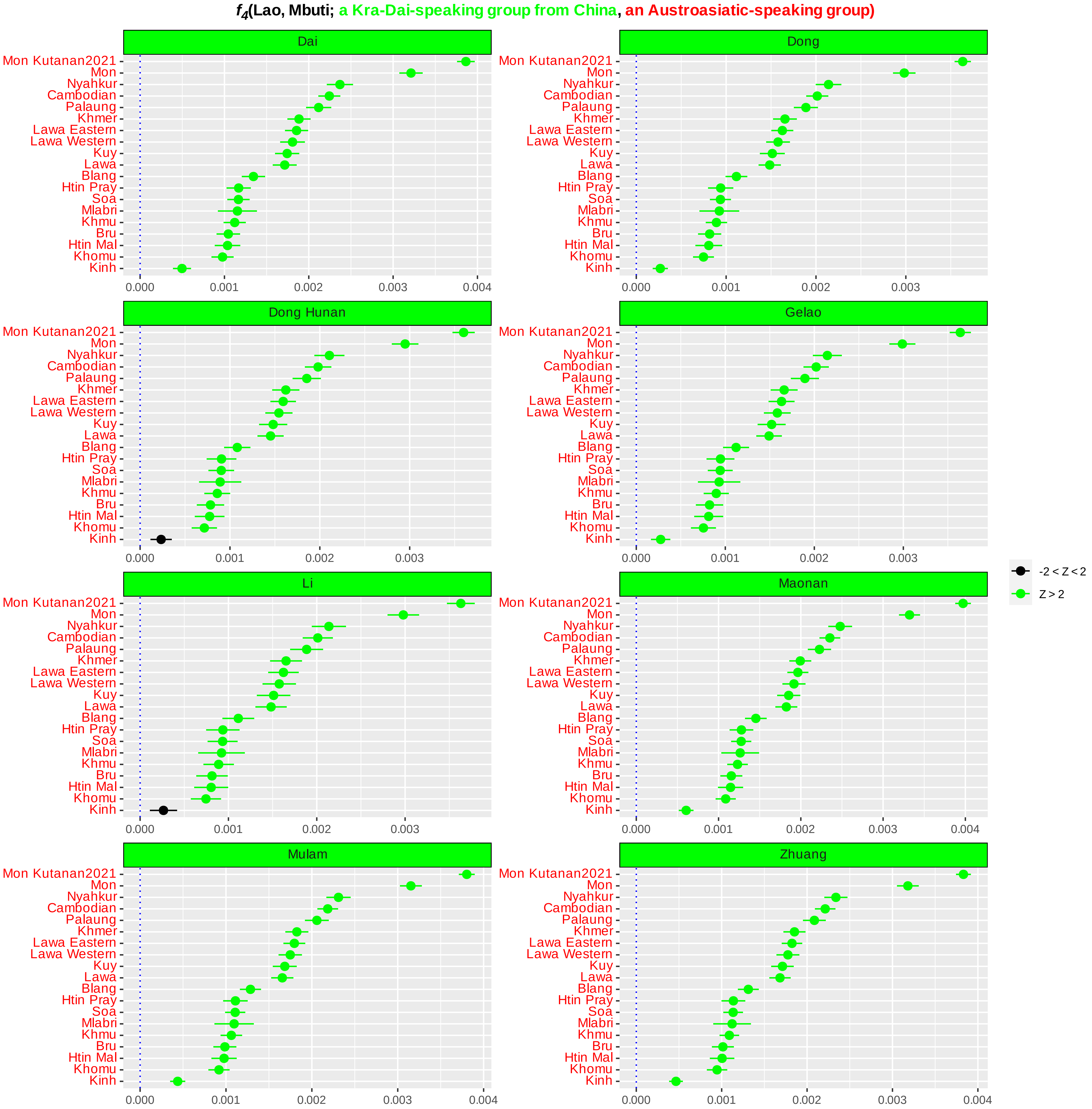

Suppl. Fig. 1. f_4-statistics of the form of f_4(Lao, Mbuti; a Kra-Dai-speaking group from China, an Austroasiatic-speaking group). A Lao from this study was used in this analysis. Kra-Dai-speaking groups are labeled above the plots on green background, while Austroasiatic-speaking groups are labeled in red. f_4-statistics with Z-scores >2 are shown in green, and those with absolute Z-scores <2 are shown in black.
