# Supplementary material for: Reanalyzing the genetic history of Kra-Dai speakers from Thailand and new insights into their genetic interactions beyond Mainland Southeast Asia
Source: Sci Rep. 2023 May 24;13:8371. doi: 10.1038/s41598-023-35507-8 (PMC10209056; doi:10.1038/s41598-023-35507-8)
Supplement: Supplementary file 3 — Supplementary Figure 3. [file 41598_2023_35507_MOESM3_ESM.pdf]

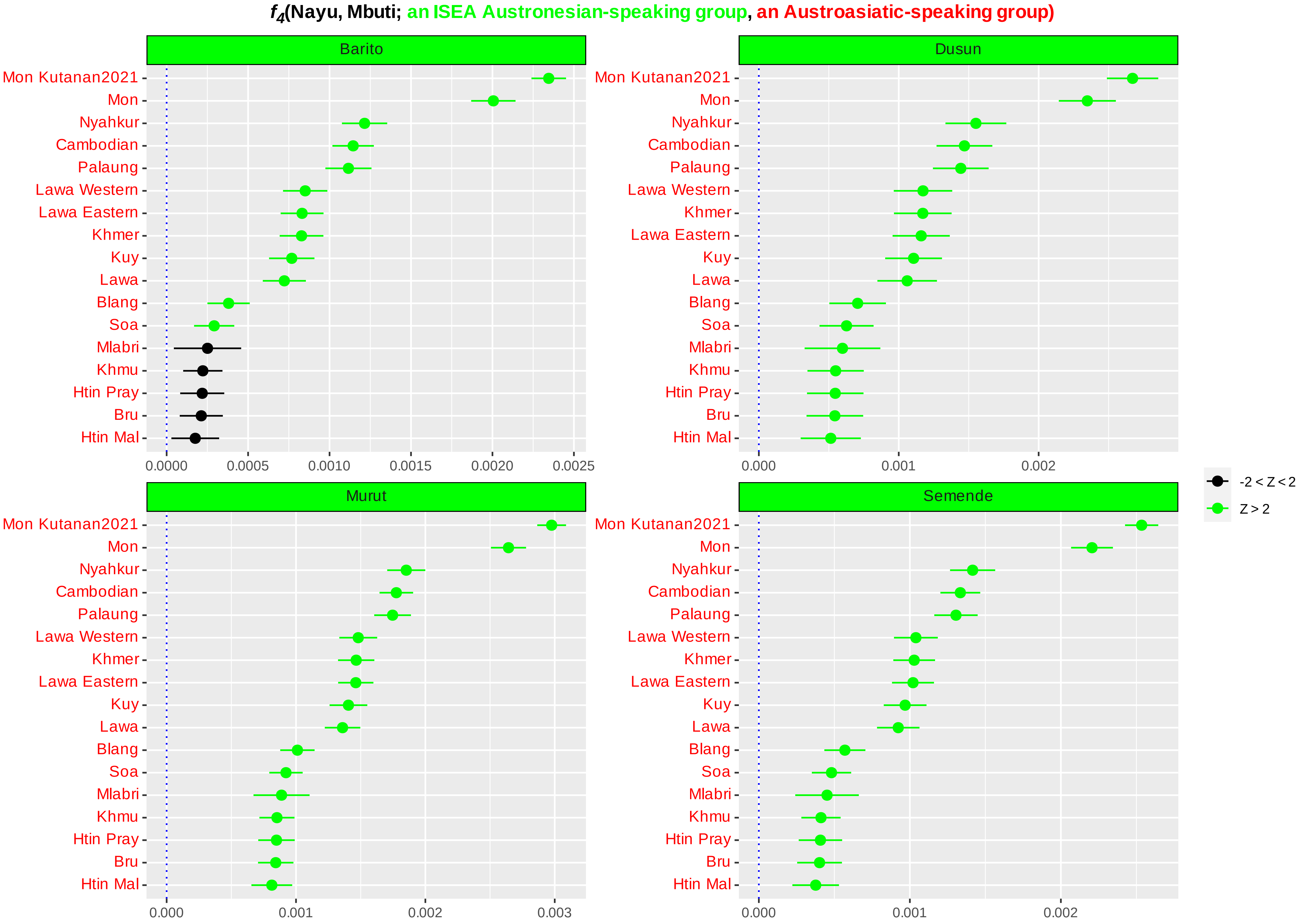

Suppl. Fig. 3. f_4-statistics of the form of f4(Nayu, Mbuti; an ISEA Austronesian-speaking group, an Austroasiatic-speaking group). ISEA Austronesian-speaking groups are labeled above the plots on green background, while Austroasiatic-speaking groups are labeled in red. f_4-statistics with Z-scores >2 are shown in green, and those with absolute Z-scores <2 are shown in black.
