# Supplementary material for: Reanalyzing the genetic history of Kra-Dai speakers from Thailand and new insights into their genetic interactions beyond Mainland Southeast Asia
Source: Sci Rep. 2023 May 24;13:8371. doi: 10.1038/s41598-023-35507-8 (PMC10209056; doi:10.1038/s41598-023-35507-8)
Supplement: Supplementary file 5 — Supplementary Figure 5. [file 41598_2023_35507_MOESM5_ESM.pdf]

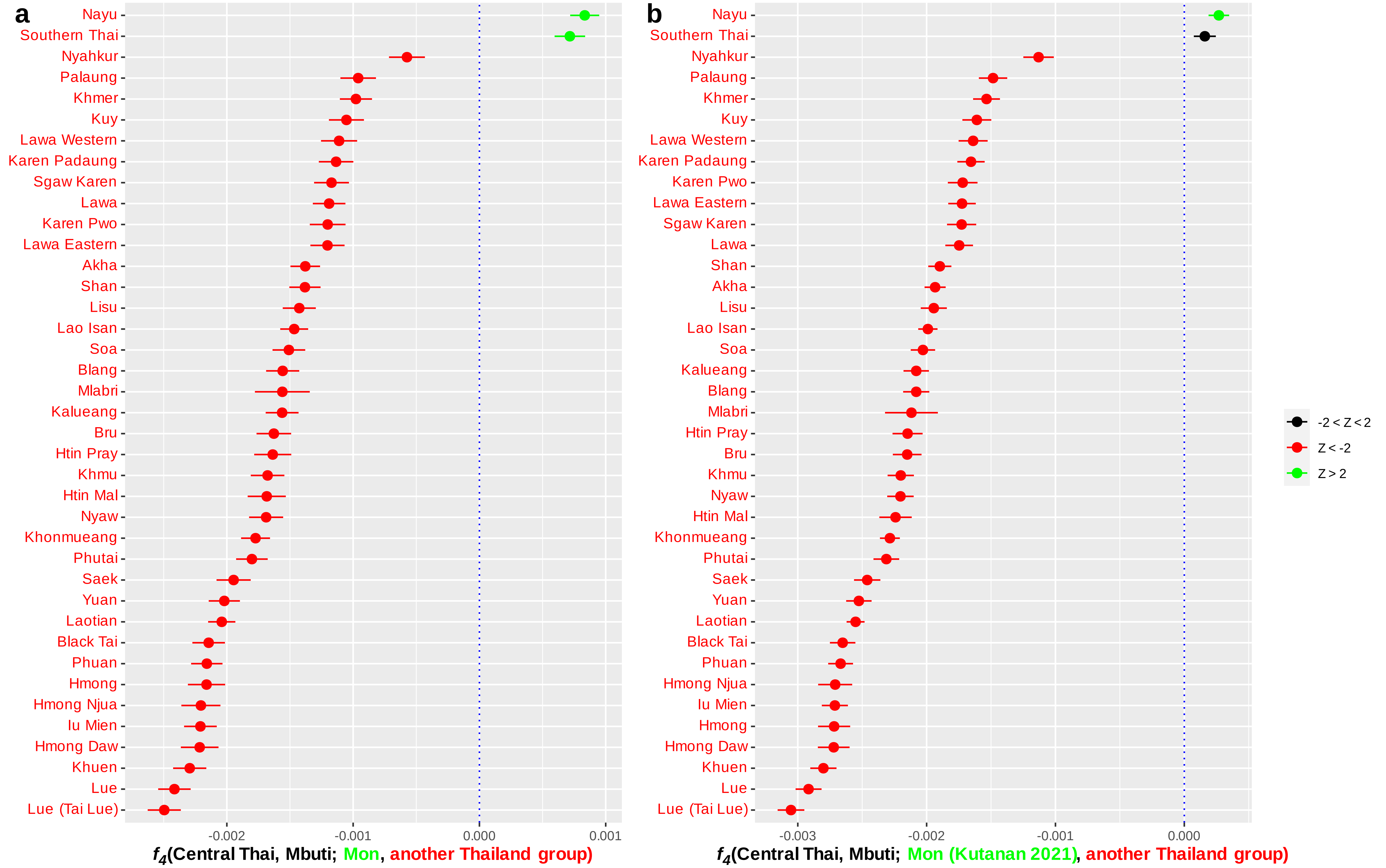

Suppl. Fig. 5. f_4-statistics of the form of f_4(Central Thai, Mbuti; Mon, another group from Thailand). Data for Mon in panels a and b are from Changmai et al. (2022)[6] and Kutanan et al. (2021)[5], respectively. f_4-statistics with Z-scores >2 are shown in green, those with Z-scores <-2 are shown in red, and those with absolute Z-scores <2 are shown in black.
